# Are children on track with their routine immunization schedule in a fragile and protracted conflict state of South Sudan? A community-based cross-sectional study

**DOI:** 10.1186/s12887-022-03213-5

**Published:** 2022-03-21

**Authors:** Israel Oluwaseyidayo Idris, Janet Tapkigen, Germaine Kabutaulaka, Gabriel Omoniyi Ayeni, Francis Ifeanyi Ayomoh, Justin Geno Obwoya

**Affiliations:** 1 Department of Population Health, London School of Hygiene and Tropical Medicine, London, UK; 2 Department of Health Sciences, University of Tampere, Tampere, Finland; 3 Department of Epidemiology and Biostatistics, New York University, New York, USA; 4 Department of Field Operation and Project Coordination, Health Pooled Fund, Rumbek, South Sudan; 5 Nuffield Department of Primary Care Health Sciences, University of Oxford, Oxford, UK

**Keywords:** Vaccine-preventable diseases, Immunization defaulters, Immunization in practice, Community healthcare, Conflict setting, South Sudan

## Abstract

**Objectives:**

The objective of this study was to assess if children aged 0–23 months in a conflict-affected state of South Sudan were on track with their immunization schedule and to identify predisposing factors that affected this study population from being on track with their routine immunization schedule.

**Design:**

Community-based cross-sectional study using a semi-structured questionnaire. The binary outcome of interest was defined as being on or off track with routine vaccination schedule. Multivariable logistic regression was used to analyze for the association between the predisposing factors surveyed and being off track with one’s routine immunization schedule.

**Setting:**

Rural communities in four counties (Rumbek Center, Rumbek North, Rumbek East and Wulu) of the Western Lakes state in South Sudan during January 10, 2020 to June 10, 2020.

**Participants:**

We surveyed 428 children aged 0–23 months and their mothers/caregivers who lived in either of the four counties in the Western Lakes State. Participants were selected using random ballot sampling.

**Results:**

More than three-quarters of the children surveyed (75.5%) were off track with their vaccination schedule. Children with an immunization card had 71% reduced odds of being off track with their immunization (AOR = 0.29; 95% CI 0.10–0.83, *p*-value = 0.021) compared to children without immunization cards. Children who reside near health facilities and do not require transportation to facilities had 87% reduced odds of being off track with their immunization compared to those who lived far and required transport to facilities. Giving an adequate immunization notice before conducting immunization outreach visits to communities was also associated with reduced odds (AOR = 0.27; 95% CI 0.09–0.78. *p*-value = 0.016) of children being off track with their immunization.

**Conclusion:**

This study revealed that most children were off track with their vaccination schedule in South Sudan, which is not only influenced by maternal characteristics but mainly by community- and state-level immunization service delivery mechanisms. Policies and interventions to improve child immunization uptake should prioritize these contextual characteristics.

**Supplementary Information:**

The online version contains supplementary material available at 10.1186/s12887-022-03213-5.

## Key points

**Question:** While there is a lot of investment in immunization in the humanitarian setting, this study is aimed at these research questions - Are children in conflict-prone regions of South Sudan on track with their immunization schedule? And what are the predisposing factors that influence immunization uptake in children aged 0–23 months living in this region and could prevent them from being on track with their routine immunization schedule?

**Finding:** This study revealed that most children in South Sudan were not on track with their vaccination schedule This finding is not only influenced by maternal characteristics but also by immunization governance at community- and state-levels.

**Meaning:** This study adds to the existing evidence that is useful to advance improvements of immunization uptake and coverage for children living in armed conflict settings.

## Introduction

Since 2012, total immunization coverage in South-Sudan has been around 47.5%, and till date, the country has remained a conflict-prone region [1–3]. Data from the WHO vaccine-preventable diseases monitoring system shows that between 2017 and 2020, there has been an increasing prevalence of Measles and Rubella diseases in the country [3, 4]. The Measles outbreak in South Sudan and the increasing prevalence of vaccine-preventable diseases could be linked to the challenges with immunization access and its cold chain logistics [5]. These challenges are linked to the South Sudanese Civil War which lasted between December, 2013 and February 2020, resulting in the vandalization and looting of about 50% of the cold-chain infrastructure in the country [6]. The low coverage of vaccine-preventable diseases in South Sudan is also related to the fragile health system [1], which is bedeviled with the several challenges ranging from health workforce shortages, poor public funding of the health system, limited access to healthcare services, as well as weak vaccine surveillance and monitoring systems [7]. The upsurge in some vaccine-preventable diseases clearly indicates the need for more attention to be given to immunization in South Sudan in a bid to forestall a resultant increase in childhood mortality that could ensue. The re-emergence and increasing prevalence of vaccine-preventable diseases in some parts of South Sudan is similar to the situation in conflict-prone regions of countries such as Iraq, Syria and Yemen [8].

Arguably, children in conflict-prone regions rightfully deserve to be fully vaccinated like their peers in other parts of the world. A significant proportion of children would need to be immunized over a prolonged period for herd immunity to be attained in their region or country, and as such, the risk of a child contracting a vaccine-preventable disease is higher if more children have either not been immunized or received incomplete immunisation [9].

In accordance with global recommendations for increased vaccination coverage of at least 90% in all countries, it is imperative that vaccination coverage and completion rates in South Sudan are given high attention to ensure that the factors impeding complete vaccination coverage are identified and novel strategies employed to bridge the gap in immunization coverage [10]. This study seeks to assess if children in a conflict-prone state of South Sudan are on track with their immunization schedule and also to identify predisposing factors that could have prevented children aged 0–23 months from being on track with their routine immunization schedule.

### Vaccination Programmes in South Sudan

South Sudan has an elaborate immunization schedule (see Additional file 1: Table 1) which stipulates that children receive a dose of Bacille Calmette-Guérin vaccine (BCG) – given at birth or first encounter with the health system, a three-dose course of the pentavalent vaccine – given at 6, 10 and 14 weeks or at least 4 weeks apart, four-doses of oral polio vaccine (OPV; given at birth, 6, 10 and 14 weeks), a dose of inactivated polio vaccine (IPV) – given at 14 weeks – and a dose of measles-containing vaccine (MCV1; administered at 9 months) [11]. In South Sudan, county level vaccination programs are developed using the micro-planning approach. Each health facility maps its health service delivery catchment areas, and groups the localities under fixed, outreached and mobile immunisation plans. Consultations are thereafter made with the stakeholders at each of the mapped localities to agree on specific day(s) for outreach or mobile sessions. Over the past years, governments, donors and partners have stepped up support to increase cold chain equipment across several counties in the country, as part of an effort to boost immunisation coverage including at fixed sites. However, some of these efforts were negatively impacted by conflicts which resulted in some of the facilities and cold chain equipment being destroyed. Furthermore, the challenge of maintenance/replacement of old equipment and the limited access to health facilities due to long distance has impacted vaccination uptake and coverage. Vaccination coverage is increased through outreach sessions, with facilities having between 4 to 16 outreach (posts) sessions in a month depending on the size of the community and its distance to catchment localities. With the hope of gradual and sustained return of peace to the country, it is anticipated that the immunization coverage target of 90% can be attained [12], especially through the use of immunisation service integration and defaulters’ information tracking systems which would foster an increase in the number of vaccination given during fixed sessions. The attainment of the global target of 90% vaccination coverage in South Sudan will require the prioritization of immunization by the government and continued support for immunization from GAVI, UNICEF, WHO and other donors/partners supporting immunization in the country. It would also require the continuation of the policy of free childhood vaccination in South Sudan [13, 14].

## Methods

### Study setting

**Fig. 1:**
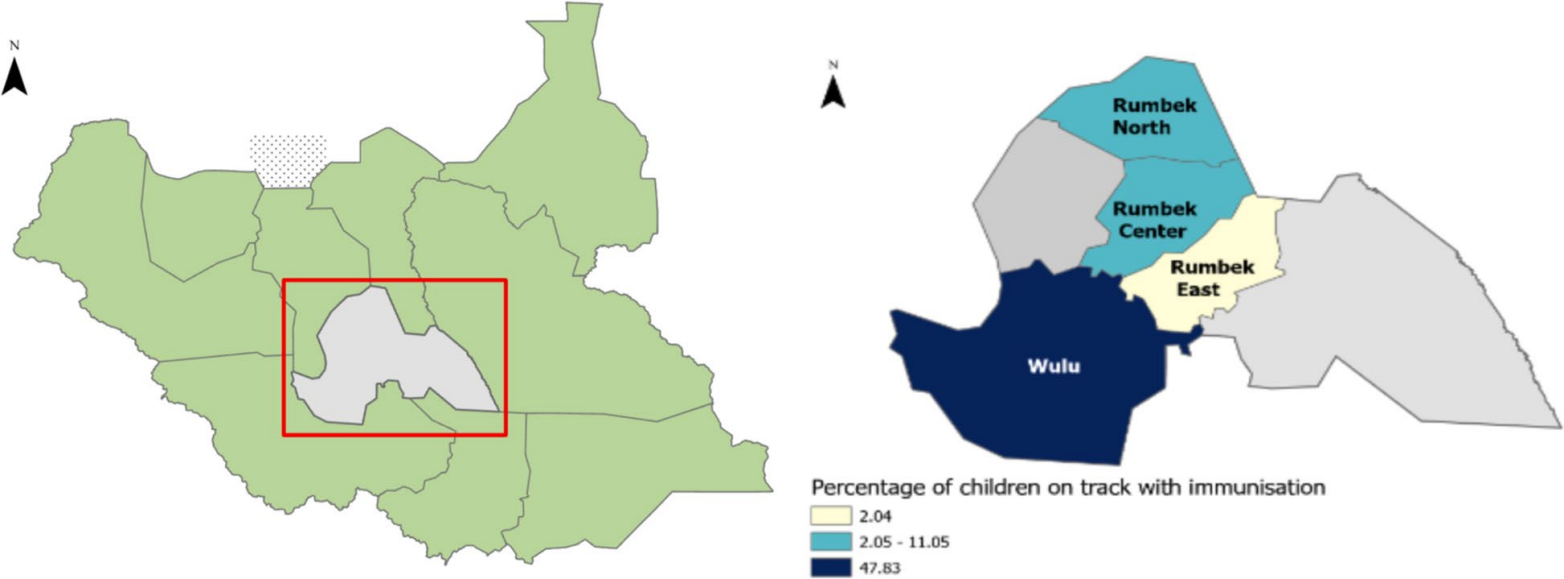
This figure contains 2 maps of the former Western Lakes state. The left map shows the geography location of the former Western Lakes in South Sudan and its four counties - Rumbek Centre, Rumbek North, Rumbek East, and Wulu. The Western Lakes State is known to be an area of protracted conflict, affecting health related outcomes including immunisation timeliness. The second map (right) shows the percentage of the sampled children aged 0–23 months living in the 4 counties in the Western Lakes State of South Sudan who were on track with their routine immunisation schedule during the survey

**Fig. 2:**
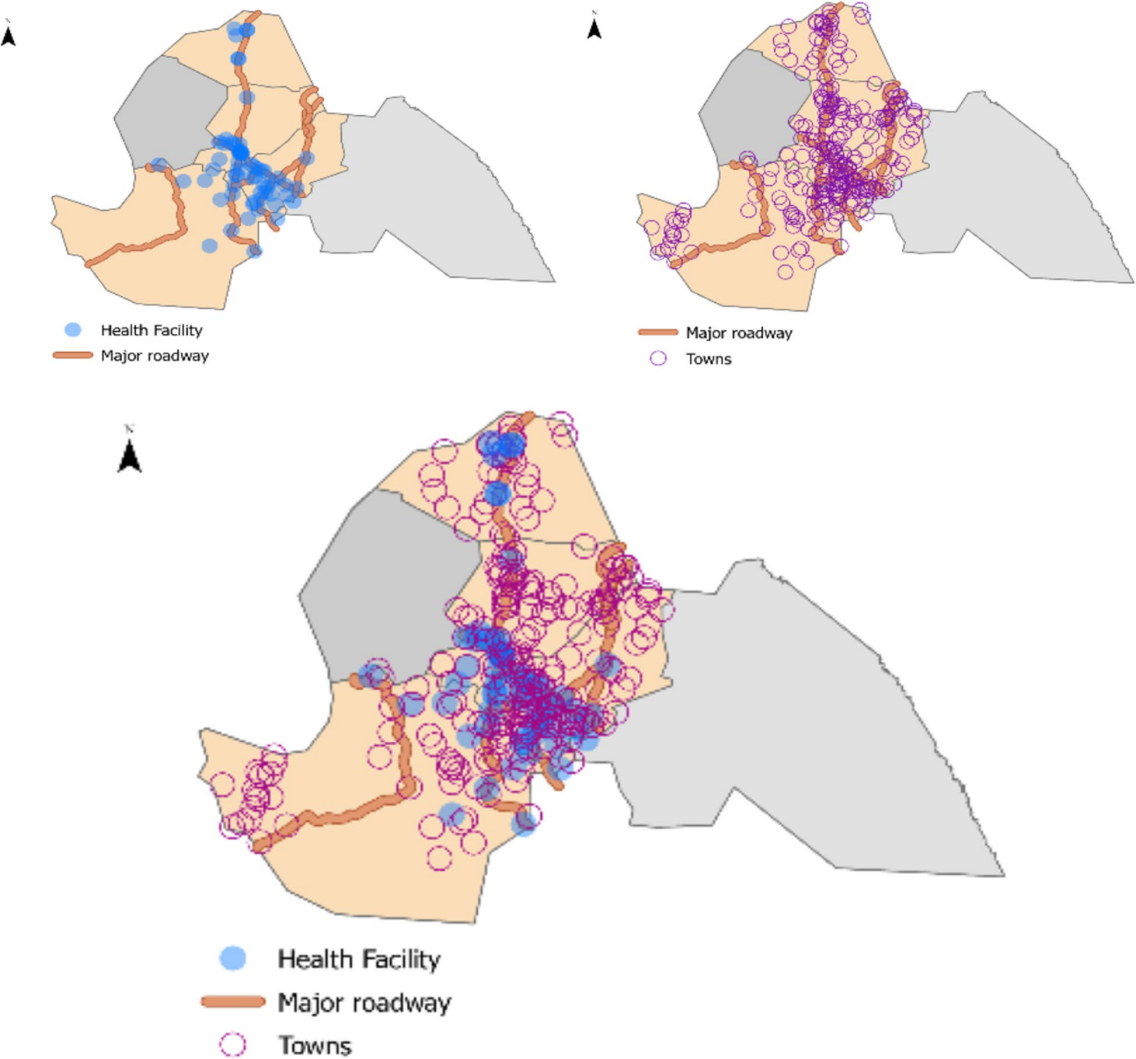
The figure has three maps showing the major roadway, towns, and health facilities in the four counties (Rumbek North, Rumbek Centre, Rumbek East and Wulu) of the Western Lakes state

This study was conducted in the rural communities of the four counties - Rumbek Center, Rumbek North, Rumbek East, and Wulu in the former Western Lakes State (see Figs. 1 and 2). The former Western Lakes state was a state in South Sudan that was abrogated to function as a state during the peace agreement signed on the 22nd of February 2020, with an aim to reduce the former 28 states to the current 10 states [15, 16]. Rumbek East, Wulu, Rumbek North and Rumbek Center counties had a population of 186,412; 61,084; 65,297 and 232,752 respectively in the July 2017 projected population estimates [17].

Since 2005, the former Western Lakes state region, one of the most insecure areas of South Sudan has experienced yearly series of armed conflict and resultant displacement, with varying factors responsible for the conflicts. From land, agricultural and water disputes to armed robberies, these violent occurrences have sustained the insecurity in South Sudan [18]. This has been worsened by re-occurrence of natural disasters e.g. heavy (destructive) flooding during the wet season [19].

### Study design

The study is a comparative community-based cross-sectional study to determine the predisposing factors for on track and off track immunization among children between 0 and 23 months in South Sudan. The study was conducted in the communities, also called bomas of the four counties aforementioned using a semi-structured questionnaire. The binary outcome was defined as whether children aged 0–23 months were on track with their immunization schedule or off track. Being on track was defined as when a child who was eligible for vaccination had received the recommended vaccines’ doses they were due for at the time of the study survey. ‘Off track’ was defined as a child who had not received a vaccine they were eligible for on or after 28 days of delay according to South Sudan’s recommended schedule [20].

### Sampling and sample size determination

We sampled 428 children and their mothers/caregivers from the sample frame using random ballot sampling.

The minimum sample size of children under 24 months required for this study was determined using the Leisle Kish formula [21, 22], with the assumption margin of 5% margin of error (d), 95% confidence interval level (Z) and the immunisation coverage assumed as 56.5% [23] taken from a study done in Juba on missed immunisation. The minimum required sample was calculated as follows:

$$\mathrm{N}=\frac{{\left({\mathrm{Z}}_{\upalpha /2}\right)}^2\mathrm{pq}}{{\mathrm{d}}^2}$$

Where:

N = the minimum sample size.

Zα/2 = the standard normal deviate corresponding to a level of significance of 0.05 is 1.96.

P = proportion of vaccine uptake or immunization defaulters or immunization refusal.

q = 1-p.

d = precision (5%);

Applying the formula, the minimum sample size is:

$$\mathrm{N}=\frac{(1.96)^2\left(0.565\ast 0.435\right)}{(0.05)^2}$$

*N* = 377.

A 13.5% increase was added to the above calculated minimum required sample size of 377, this was to adjust for non-responses and invalid responses. Although, all of the 428 respondents participated in the study.

### Data collection procedure

Data collection was carried out by three integrated community case management (ICCM) workers per boma who volunteered and collected data between January 10, 2020 and June 10, 2020. The volunteers were given a 2-day orientation on administering the questionnaire. The questionnaire was adopted from the demographic and health survey of Ethiopia [24] and the WHO’s questionnaire [25, 26], with slight modifications based on the study site context. Volunteers were monitored by a supervisor to ensure compliance with data collection guidelines and they filled the answers themselves for uniformity, although respondents were also allowed to read the survey questionnaire for proper/additional understanding of the questions. The questions were translated in the dinka language by certified translators and the community chiefs confirmed that the translation was correct. The questionnaire was simple, with “yes” or “no” responses, an approach taken to ensure that the level of education of the mothers did not significantly influence the answers. Survey respondents were mothers and caregivers above 16 years-old with children aged 0–23 months. Immunization history of a child was primarily obtained from their respective immunization cards; wherein if lost or unavailable, immunization history was obtained from the mothers’/caregivers’ and verified from the immunization register at the health center where the immunization was taken, with the exception for BCG vaccination for which the scar proved as evidence. Children with untraceable immunization records were excluded from the study..

### Statistical analysis

The primary quantitative data was entered into Microsoft Excel and imported into STATA 15 for analysis. Missing data was observed and accounted for in the analysis and reported in the tables. Maternal age was regrouped into below 24 years, 25–34 years and above 35 years. Age of the child was grouped into 0–11 months and 12–23 months. Birth order of the child was categorized into 1st child, 2nd-5th order and 6th + order. Maternal education was defined as none and having some education. Sex of the child was defined as female and male. Distance to the health facility was categorized into <=5Km, 6-10KM and > =11 Km. Four approaches were used to analyze the data. First, we performed descriptive analyses to compute the frequencies and percentages of the variables. Second, crude odds ratio (cOR) with 95% confidence intervals (CI) were estimated using univariate logistic regression to quantify the relationship between the outcome with the independent variables. The univariate analysis was used to identify factors that were independently associated with the outcome. Third, factors that were statistically associated with the outcome in the univariate analysis were treated as main exposures and adjusted for potential confounders using the Mantel-Haenszel method. Crude odds ratio from the univariate analysis was compared with the Mantel-Haenszel’s OR to identify potential confounders. Homogeneity testing was conducted to compare stratum specific odds ratios in order to identify effect modification. Lastly, the potential confounders were entered into a multi-level multivariable and forward selection stepwise multivariable logistic regression models to control for them. Factors treated as main exposures included in the models included parity, frequent provision of immunisation at health facility on daily basis, immunisation benefits, vaccinator conducting outreach immunisation, vaccinator visiting during family livelihood/ business hours, health facility is too far and no transportation means to visit health facility for immunisation, inadequate notice about immunisation outreach visit to community, churches discussed immunisation importance, community leaders discussed immunisation importance. Prior to fitting the multivariable models, we assessed for collinearity using the Pearson’s R correlation coefficient (r > =0.8), and retained predictors that were within the recommended range as suggested in the literature [27].

Variables with collinearity include religion, marital status, fear of side effects and abusive vaccinators. These variables had all or very few observations in one group, thus, no variations. Estimates with a *p*-value less than significance level (5%) were considered statistically significant. Odds ratios (OR), *p*-values and lower and upper 95% confidence intervals of the variables that were included in the model are presented.

### Ethical consideration

Ethical approval for the study was obtained from the Ministry of Health in South Sudan. Permission to conduct the study was obtained from the state manager of the extended programme on immunisation and the director of primary healthcare, state ministry of health. Prior to completing the questionnaires, participants were given information about the study and written informed consent was obtained. Participants were informed of their right to voluntary participation in the study.

## Results

### Participant characteristics

Four hundred and twenty eight mothers and caregivers of children aged 0–23 months were included in this study. The median age of the children in the study was 10 months, with 64% (274/428) being 0–11 months and 52.0% (222/427) being boys. Mothers made up 76.5% of the respondents (313/409) compared to caregivers. The median age of the mothers and caregivers was 28 years (age range 15–52) with a majority of the mothers and caregivers (48.6%) between 25 and 34 years. Majority of the respondents (78.7%) had no formal education. Mothers with 2–5 children formed the largest proportion (63.4%) of participants. Over half (55.2%) of the children reported having immunization cards.

### Immunisation status

Overall, findings from this study showed that 75.5% (320/424) of the children were off track with immunisation (Table 1). There was no difference in the proportion of children being off track with immunisation (*p* value = 0.152) between female children (78.3%) and male children (72.3%). Compared to the rest of the recommended vaccination doses, BCG had the least proportion of children being off track with immunisation (12.2%, 52/428). For vaccines with more than one dose, such as Penta and OPV, the proportion of children being off track with immunisation increased in subsequent immunisation doses than preceding doses. For instance, while only 19.4% (80/413) of children were off track with the 1st dose of the pentavalent vaccination, more than half (60.6%, 215/355) of the children were off track with the 3rd dose of the pentavalent vaccination.

**Table 1:** Distribution of the proportion of vaccine coverage by vaccine and gender

Variable	Overall on track with immunization	On track with immunization by Gender	
	Frequency ***N*** (%)	Male ***N*** (%)	Female ***N*** (%)
**BCG vaccine**			
Off track	52 (12.15)	26(11.71)	26 (12.68)
On track	376 (87.85)	196(88.29)	179 (87.32)
**1st dose of OPV and Pentavalent vaccine**			
Off track	80 (19.37)	40(18.02)	41 (20.00)
On track	333 (80.63)	182 (81.98)	164 (80.00)
**2nd dose of OPV and Pentavalent vaccine**			
Off track	135 (35.62)	74 (37.56)	61 (33.70)
On track	244 (64.38)	123 (62.44)	120 (66.30)
**3rd dose of OPV and Pentavalent vaccine**			
Off track	215 (60.56)	114 (61.29)	100 (59.52)
On track	140 (39.44)	72 (38.71)	68 (40.48)
**IPV**			
Off track	126 (35.59)	68 (36.56)	58 (34.73)
On Track	228 (64.41)	118 (63.44)	109 (65.27)
**Measles vaccine (9–23 months)**			
Off track	180 (70.87)	96 (71.64)	84 (70.00)
On track	74 (29.13)	38 (28.36)	36 (30.00)
**Child on/off track with immunization status**			
Children on track with immunization	104 (24.53)	48 (21.72)	56 (27.72)
Children off track with immunization	320 (75.47)	173 (78.28)	146 (72.28)

### Factors associated with being off track with immunisation

In the univariate analysis (see Table 2), there was a strong statistical association between being off track with immunisation and being in Wulu County (crude odds ratio (cOR) 0.13, 95% CI: 0.07–0.23); mothers with 6 and above children (cOR 0.42, 95% CI: 0.19–0.93); being a guardian (cOR 6.32, 95% CI: 2.67–14.96); maternal age 25–34 years (cOR 1.98, 95% CI: 1.17–3.36); immunisation card unavailable (COR 3.49, 95% CI 2.09–5.81); functional facility being a Primary Health Care Center (cOR 4.63, 95% CI: 2.59–8.25); health facility does not conduct routine immunisation outreach (cOR 2.61, 95% CI: 1.50–4.51); no frequent provision of (EPI) immunisation at health facility (cOR 1.80; 95% CI: 1.11–2.90); Immunisation benefits being child growth (cOR 3.96, CI 95% (1.35, 11.53); no vaccinator conducting outreach immunisation (cOR 2.32, CI 95% 1.45–3.71); vaccinator comes during family livelihood business time (cOR 0.39, 95% CI 0.21–0.68); for health facility in close proximity with transportation (cOR 0.20, 95% CI 0.10–0.37); vaccinator are happy to regularly come to the community (cOR 0.13, 95% CI 0.04–0.43); completed all immunization for child and informed to come back (cOR 0.37, 95% CI 0.19, 0.71); Adequate notice about immunization outreach visit to community (cOR 0.23, 95% CI 0.12–0.42); boma health workers visiting household 4–14 times (cOR 0.16 95% CI 0.07–0.36); churches not discussing immunisation importance (cOR 7.42, 95% CI 4.28–12.82); and community leaders not discussing immunization importance (cOR 7.76 95% CI 4.50–13.36). In addition, there were potential confounding effects on the above associations. For example, and as seen in Table 3, after adjusting for maternal age, children whose mothers and caregivers had adequate notice about immunization outreach were about 90% less likely to be off track with immunization compared to those who did not receive adequate notice (cOR 0.11, 95% CI 0.04–0.29).

**Table 2:** Descriptive and univariate analysis for predisposing factors that affected children between 0 and 23 months old living in South Sudan from being on track with their routine immunisation schedule

Variable	Category	On track ***N*** (%)	Off track ***N*** (%)	Crude Odds Ratio	
				OR and (95% CI)	***P***-value
**Geographical Characteristics**					
**County (** ***N*** **= 427)**	Rumbek centre	20 (19.23)	155 88.57)	1	
	Rumbek north and Rumbek east	3 (9.23)	78 (96.30)	3.35 (0.96, 11.63)	0.056
	Wulu	81 (48.50)	86 (51.50)	0.13(0.07, 0.23)	**< 0.001**
**Maternal Characteristics/Role**					
**Parity (** ***N*** **= 418)**	1 child	11 (19.30)	46 (80.70)	1	
	2–5 children	59 (22.52)	203 (77.48)	0.82 (0.40, 1.68)	0.595
	6 and above	34 (35.79)	61 (64.21)	0.42 (0.19, 0.93)	**0.034**
**Education of Mothers (** ***N*** **= 422)**	None	77 (23.33)	253 (76.67)	1	0.444
	Having some education	24 (27.27)	64 (72.73)	0.81 (0.47,1.38)	
**Are you Parent/guardian of the child (** ***N*** **= 409)**	Parent	94 (30.13)	218 (69.87)	1	**< 0.001**
	Guardian	6 (6.38)	88 (93.62)	6.32(2.67, 14.96)	
**Maternal age (** ***N*** **= 385)**	< 24 years	39 (33.33)	78 (66.67)	1	
	25–34 years	37 (20.11)	147 (79.89)	1.98 (1.17, 3.36)	**0.011**
	> 35 years	17 (21.25)	63 (78.75)	1.85 (0.95, 3.58)	0.067
**Availability of child immunisation card (** ***N*** **= 411)**	Yes	78 (34.67)	147 (65.33)	1	**< 0.001**
	No	24 (13.19)	158 (86.81)	3.49 (2.09, 5.81)	
**Child Characteristics**					
**Sex (** ***N*** **= 427)**	Male	48 (21.72)	173 (78.28)	1	0.153
	Female	56 (27.72)	146 (72.28)	0.72 (0.46, 1.12)	
**Child age (** ***N*** **= 428)**	0–11 Months	71 (26.10)	201 (73.90)	1	0.314
	12–23 Months	33(21.71)	119 (78.29)	1.27 (0.79, 2.04)	
**Communal/Health System Role**					
**Functional health facility in the area (** ***N*** **= 420)**	Yes	99 (78.29)	285 (74.22)	1	0.80
	No	3 (10.34)	26 (89.66)	1.86 (0.40, 8.54)	
**Functional facility type in the area (** ***N*** **= 413)**	PHCU	86 (34.26)	165 (65.74)	1	**< 0.001**
	PHCC	16 (10.13)	142 (89.87)	4.63 (2.59, 8.25)	
**Distance to facility** ***N*** **= (406)**	<=5 km	39 (25.49)	114 (74.51)	1	0.082
	6-10Km	22 (18.80)	95 (81.20)	1.47 (0.81, 2.66)	
	> = 11Km	41 (31.06)	91(68.94)	0.75 (0.45, 1.27)	
**Facility provides immunization (** ***N*** **= 417)**	Yes	99 (25.78)	285 (74.22)	1	0.076
	No	3 (10.34)	26 (89.66)	3.01 (0.89, 10.16	
**Health facility conduct routine immunization outreach services (** ***N*** **= 417)**	Yes	84 (30.11)	195 (69.89)	1	**0.001**
	No	19 (14.18)	115 (85.82)	2.61 (1.50, 4.51)	
**Frequent provision of (EPI) immunization at health facility on daily bases (** ***N*** **= 420)**	Yes	73 (28.85)	180 (71.15)	1	**0.017**
	No	30 (18.40)	133 (81.60)	1.80(1.11, 2.90)	
**Health facility Conduct mobile immunization (** ***N*** **= 418)**	Yes	56 (27.72)	146 (72.28)	1	0.192
	No	47 (22.17)	165 (77.83)	1.34 (0.861, 2.10)	
**Is immunization good for child’s health (** ***N*** **= 416)**	Yes	100 (25.25)	296 (74.75)	1	0.26
	No	2 (12.50)	14 (87.50)	2.36 (0.52, 10.58)	
**Immunization benefits (** ***N*** **= 324)**	Protection against diseases	97 (33.80)	190 (66.20)	1	**0.012**
	Child growth	4 (11.43)	31 (88.57)	3.96 (1.35, 11.53)	
**Vaccinator conduct outreach immunization (** ***N*** **= 409)**	Yes	67 (32.37)	140 (67.63)	1	**< 0.001**
	No	34 (17.09)	165 (82.91)	2.32 (1.45, 3.71)	
**Vaccinator visit during family livelihood business (** ***N*** **= 408)**	Yes	17 (14.05)	104 (85.95)	1	**0.001**
	No	84 (29.58)	200 (70.42)	0.39 (0.21, 0.68)	
**Health facility too far and no transportation to going for immunization (** ***N*** **= 410)**	Yes	13 (9.15)	129 (90.85)	1	**< 0.001**
	No	89 (33.58)	176 (66.42)	0.20(9.10, 0.37)	
**Vaccinator ask for money when visit the facility for services (** ***N*** **= 406)**	Yes	3 (33.33)	6 (66.67)	1	0.552
	No	97 (24.62)	297 (75.38)	1.53(0.37, 6.23)	
**Vaccinator not happy for coming regularly to the community (** ***N*** **= 407)**	Yes	3 (5.08)	56 (94.92)	1	**0.001**
	No	99 (28.70)	246 (71.30)	0.13(0.04, 0.43)	
**Not completed all the immunization for my child, not informed to come back (** ***N*** **= 405)**	Yes	12 (13.04)	80 (86.96)	1	**0.003**
	No	89 (28.71)	221 (71.29)	0.37(0.19, 0.71)	
**Inadequate notice about immunization outreach visit to community (** ***N*** **= 408)**	Yes	13 (9.85)	119 (90.15)	1	**< 0.001**
	No	88 (32.23)	185 (67.77)	0.23 (0.12, 0.42)	
**Girl child don’t receive immunization if yes why (** ***N*** **= 408)**	Yes	3 (30.00)	7 (70.00)	1	0.709
	No	98 (24.81)	297 (75.19)	1.29 (0.32, 5.11)	
**Male child don’t receive immunization if yes why (** ***N*** **= 409)**	Yes	3 (33.33)	6 (66.67)	1	0.556
	No	98 (24.69)	299 (75.31)	1.52(0.374, 6.21)	
**Relocation history in the past 23 months (** ***N*** **= 400)**	Yes	19 (29.69)	45 (70.31)	1	0.395
	No	82 (24.62)	251 (75.38)	1.29(0.71, 2.33)	
**Times BHW visit household (** ***N*** **= 345)**	No visitation	10 (13.70)	63 (86.30)	1	**< 0.001**
	1–3 times	24 (13.87)	149 (86.13)	0.98(0.44, 2.18)	
	4–14 times	47 (48.96)	49 (51.04)	0.16(0.07, 0.36)	
**Churches discussed immunization importance (** ***N*** **= 373)**	Yes	71 (44.65)	88 (55.35)	1	**< 0.001**
	No	21 (9.81)	193 (90.19)	7.42(4.28, 12.82)	
**Community leaders discussed immunization importance (** ***N*** **= 374)**	Yes	70 (46.05)	82 (53.950	1	**< 0.001**
	No	22 (9.91)	200 (90.09)	7.76(4.50, 13.36)

**Table 3:** Adjusted estimates of the odds ratio for the main exposures influencing children between 0 and 23 months old living in South Sudan from being on track with their routine immunisation schedule using Mantel-Haenszel method

Variables	Crude Estimates		Adjusted Estimates	
	Crude OR (95%CI)	***P***-value	Adjusted OR (95%CI)	***P***-value
**Parity: Association adjusted for:**				
Maternal Age (25–34 years)	0.42 (0.19, 0.93)	0.034	0.40 (0.24, 0.65)	< 0.001
**Immunization benefits adjusted for:**				
Maternal Age (25–34 years)	3.96 (1.35, 11.53)	**0.012**	3.82 (1.25, 11.60)	0.011
Frequent provision of immunization at health facility on daily bases			3.50(1.18, 10.34)	0.016
Health facility too far and no transportation to going for immunization			3.95 (1.27, 12.23)	0.01
**Frequent provision of immunization at health facility on daily basis adjusted for:**				
County	1.80(1.11, 2.90)	0.017	1.20 (0.70, 2.03)	0.494
Availability of children immunization card			1.62 (0.97, 2.69)	0.059
Functional health facility in the area			1.64 (0.98, 2.73)	0.054
Health facility conduct routine immunization outreach services			1.33 (0.77, 2.27)	0.303
Health facility too far and no transportation to going for immunization			1.53 (0.92, 2.53)	0.099
Vaccinator not happy for coming regularly to the community			1.54 (0.94, 2.53)	0.083
Inadequate notice about immunization outreach visit to community			1.32 (0.78, 2.22)	0.293
**vaccinator conduct outreach immunization adjusted for:**				
Maternal Age (25–34 years)	2.32 (1.45, 3.71)	**< 0.001**	2.15 (1.27, 3.63)	0.003
immunization benefits			2.06 (1.231, 3.43)	0.005
Vaccinator visit during family livelihood business			2.23 (1.36, 3.61)	0.001
Health facility too far and no transportation to going for immunization			2.31 (1.40, 3.78)	0.001
Not completed all the immunization for my child, not informed to come back			2.26 (1.39, 3.65)	0.001
**Vaccinator visit during family livelihood business adjusted for:**				
Availability of children immunization card	0.39 (0.21, 0.68)	**0.001**	0.35 (0.19, 0.64)	0.001
Functional facility type in the area			0.35 (0.19, 0.64)	< 0.001
Health facility conduct routine immunization outreach services			0.36 (0.19, 0.66)	0.001
**Health facility too far and no transportation to going for immunization adjusted for:**				
Functional facility type in the area	0.20 (9.10, 0.37)	< 0.001	0.15 (0.07, 0.29)	< 0.001
Inadequate notice about immunization outreach visit to community			0.20 (0.10, 0.37)	< 0.001
**Inadequate notice about immunization outreach visit to community adjusted for:**				
Parity (above 6 children)	0.23 (0.12, 0.42)	**< 0.001**	0.22 (0.11, 0.42)	< 0.001
Availability of children immunization card			0.24 (0.12, 0.45)	< 0.001
Maternal Age			0.11 (0.04, 0.29)	< 0.001
Vaccinator conduct outreach immunization			0.19 (0.09, 0.36)	< 0.001
Health facility too far and no transportation to going for immunization			0.22 (0.11, 0.42)	< 0.001
Vaccinator not happy for coming regularly to the community			0.28 (0.14, 0.53)	< 0.001
**Churches discussed immunization importance adjusted for:**				
County	7.42(4.28, 12.82)	**< 0.001**	2.42 (1.15, 5.11)	0.016
Availability of children immunization card			6.90 (3.63, 13.06)	< 0.001
Functional facility type in the area			6.60 (3.46, 12.54)	< 0.001
Health facility conduct routine immunization outreach services			6.94 (3.66, 13.15)	< 0.001
Frequent provision of immunization at health facility on daily bases			6.90 (3.94, 12.09)	< 0.001
Vaccinator visit during family livelihood business			6.48 (3.56, 11.76)	< 0.001
Health facility too far and no transportation to going for immunization			6.07 (3.28, 11.21)	< 0.001
Not completed all the immunization for my child, not informed to come back			6.59 (3.58, 12.11)	< 0.001
Inadequate notice about immunization outreach visit to community			6.91 (3.78, 12.61)	< 0.001
Community leaders discussed immunization importance			2.29 (0.98, 5.35)	0.049
Are you Parent/guardian of the child			6.38 (3.26, 12.45)	< 0.001
**Community discussed immunization importance adjusted for:**				
Availability of children immunization card	7.76(4.50, 13.36)	**< 0.001**	6.43 (3.40, 12.16)	< 0.001
Functional facility type in the area			6.81 (3.50, 13.25)	< 0.001
Health facility conduct routine immunization outreach services			7.61 (3.90, 14.85)	< 0.001
Frequent provision of immunization at health facility on daily bases			7.52 (4.22, 13.41)	< 0.001
Vaccinator visit during family livelihood business			6.86 (3.78, 12.45)	< 0.001
Health facility too far and no transportation to going for immunization			6.57 (3.54, 12.16)	< 0.001
Not completed all the immunization for my child, not informed to come back			6.98 (3.78, 12.84)	< 0.001
Inadequate notice about immunization outreach visit to community			6.83 (3.68, 12.63)	< 0.001
Churches discussed immunization importance			3.14 (1.33, 7.38)	0.006
Are you Parent/guardian of the child			6.71 (3.44, 13.03)	< 0.001
Parity (above 6 children)			7.00 (3.87, 12.65)	< 0.001

### Multivariable analysis

The stepwise multivariable logistic regression models results (see Table 4) showed that the predisposing factors that mainly were associated with the study population’s immunization status was not children’s characteristics such as sex and age, but rather, by community- and state-level contexts. In fact, children were up to more than 2 times more likely to be off track with immunization when there was no vaccinator conducting outreach immunization compared to when there was (OR 2.27, 95% CI 1.29–3.96). The probability of a child being off track for immunization was reduced by 85% for those who lived close to health facilities and had transportation compared to those who did not (OR 0.15, 95% CI 0.07–0.28). Children whose mothers and caregivers had adequate notice about immunization outreach were up to 85% less likely to be off track with immunization compared to those who did not receive adequate notice (OR 0.15, 95% CI 0.07–0.28). Lastly, it was observed that the absence of community engagement discussing immunization importance in both the church (OR 2.42, 95% CI 1.16–5.01) and from the greater community (OR 3.62, 95% CI1.42–9.18) increased the odds of children being off track with immunization compared to communities that did engage.

**Table 4:** Multilevel multivariate logistics regression models to identify the predisposing factors that affected children between 0 and 23 months old living in South Sudan from being on track with their routine immunisation schedule

**Variable**	**Crude Estimate**	**Model 1a**	**Model 2b**	**Model 3c**
	**cOR (CI)**	**aOR (CI)**	**aOR (CI)**	**aOR (CI)**
Individual-level factors				
**Maternal and Child Characteristics**				
**Parity**				
1 child	1 (reference)	1 (reference)	1 (reference)	1 (reference)
2–5 children	0.82 (0.40, 1.68)	0.55 (0.23, 1.28)#	0.58 (0.18, 1.85)#	1.01 (0.45, 2.23)#
6 and above	0.43 (0.19, 0.93)	0.28 (0.09, 0.83)	0.32 (0.08, 1.12)#	0.40 (0.16, 0.96)
**Community Characteristics**				
**Immunization benefits**				
Protection against diseases	1 (reference)	1 (reference)	1 (reference)	1 (reference)
Child Growth	3.96 (1.35,11.53)	5.94 (1.70, 20.71)	23.05 (2.81,188.68)	7.45 (2.06, 26.87)
**Churches discussed immunization importance**				
Yes	1 (reference)	1 (reference)	1 (reference)	1 (reference)
No	7.42 (4.28,12.82)	5.75 (2.79, 11.80)	1.62 (0.60, 4.37)#	7.62 (3.90, 14.87)
**Community discussed immunization importance**				
Yes	1 (reference)	1 (reference)	1 (reference)	1 (reference)
No	7.76 (4.50,13.36)	5.62 (2.6, 11.86)	1.77 (0.58, 5.34)#	8.01 (4.02, 15.92)
**State (health system) related factors**				
**Frequent provision of immunization at health facility on daily bases**				
Yes	1 (reference)	1 (reference)	1 (reference)	1 (reference)
No	1.80 (1.11, 2.90)	1.93 (1.04, 3.57)	1.59 (0.80, 3.14)#	1.01 (0.53, 1.89)#
**Vaccinator conduct outreach immunization**				
Yes	1 (reference)	1 (reference)	1 (reference)	1 (reference)
No	2.32 (1.45,3.71)	2.27 (1.29, 3.98)	4.12 (1.91, 8.88)	7.21 (3.82, 13.5)
**Vaccinator visit during family livelihood business**				
Yes	1 (reference)	1 (reference)	1 (reference)	1 (reference)
No	0.40 (0.21,0.68)	0.56 (0.29,1.09)	0.32 (0.13, 0.73)	0.43 (0.23, 0.80)
**Health facility too far and no transportation to going for immunization**				
Yes	1 (reference)	1 (reference)	1 (reference)	1 (reference)
No	0.20 (0.10,0.37)	0.26 (0.12, 0.54)	0.19 (0.07, 0.48)	0.17 (0.08, 0.35)
**Inadequate notice about immunization outreach visit to community**				
Yes	1 (reference)	1 (reference)	1 (reference)	1 (reference)
No	0.23 (0.12, 0.42)	0.15 (0.05, 0.39)	0.39 (0.16, 0.91)	0.68 (0.29, 1.55)#
**Multivariable logistic regression models to control for them using forward selection stepwise regression**				
	**Crude Estimates**		**Adjusted Estimates**	
**Variables**	**Crude Odd Ratio (95% CI)**	***P*** **-value**	**Adj Odd Ratio (95% CI)**	***P*** **-value**
**Immunization benefits: Association:**				
Immunisation benefit adjusted for Frequent provision of immunization at health facility on daily bases	3.96 (1.35,11.53)	0.012	3.53 (1.199, 10.36)	0.022
Immunisation benefit adjusted for (Frequent provision of immunization at health facility on daily bases + Maternal Age (25–34 years))			3.36 (1.12, 10.05)	0.03
Immunisation benefit adjusted for (Frequent provision of immunization at health facility on daily bases + Maternal Age (25–34 years) + Health facility too far and no transportation to going for immunization)			4.34 (1.23, 15.24)	0.022
**vaccinator conduct outreach immunization**				
vaccinator conduct outreach immunization adjusted for immunisation benefit	2.32 (1.45,3.71)	< 0.001	2.05 (1.24, 3.39)	0.005
vaccinator conduct outreach immunization adjusted for (immunisation benefit + Maternal age (25–34 years old))			2.18 (1.25, 3.78)	0.005
vaccinator conduct outreach immunization adjusted for (immunisation benefit + Maternal age (25–34 years old) + Vaccinator visit during family livelihood business)			2.27 (1.29, 3.96)	0.004
vaccinator conduct outreach immunization adjusted for (immunisation benefit + Maternal age (25–34 years old) + Vaccinator visit during family livelihood business + Not completed all the immunization for my child, not informed to come back)			2.26 (1.29, 3.94)	0.004
vaccinator conduct outreach immunization adjusted for (immunisation benefit + Maternal age (25–34 years old) + Vaccinator visit during family livelihood business + Not completed all the immunization for my child, not informed to come back + Health facility too far and no transportation to going for immunization)			1.81 (1.00, 3.25)	0.046
**Vaccinator visit during family livelihood business:**				
Vaccinator visit during family livelihood business adjusted for Availability of children immunization card	0.40 (0.21,0.68)	0.001	0.35 (0.19, 0.64)	0.001
Vaccinator visit during family livelihood business adjusted for Availability of children immunization card + Functional facility type in the area)			0.32 (0.17, 0.59)	< 0.001
Vaccinator visit during family livelihood business adjusted for Availability of children immunization card + Functional facility type in the area + Health facility conduct routine immunization outreach services			0.33 (0.17, 0.62)	0.001
**Health facility too far and no transportation to going for immunization:**				
Health facility too far and no transportation to going for immunization adjusted for Functional facility type in the area	0.20 (0.10,0.37)	< 0.001	0.14 (0.07, 0 .26)	< 0.001
Health facility too far and no transportation to going for immunization adjusted for Functional facility type in the area + Inadequate notice about immunization outreach visit to community)			0.15 (0.07, 0.28)	< 0.001
**Inadequate notice about immunization outreach visit to community:**				
Inadequate notice about immunization outreach visit to community adjusted for Maternal Age	0.23 (0.12, 0.42)	< 0.001	0.11 (0.04, 0.28)	< 0.001
Inadequate notice about immunization outreach visit to community adjusted for Maternal Age + vaccinator conduct outreach immunisation			0.10 (0.03, 0.25)	< 0.001
Inadequate notice about immunization outreach visit to community adjusted for Maternal Age + vaccinator conduct outreach immunisation + Parity (above 6 children)			0.10 (0.03, 0.25)	< 0.001
Inadequate notice about immunization outreach visit to community adjusted for Maternal Age + vaccinator conduct outreach immunisation + Parity (above 6 children) + Health facility too far and no transportation to going for immunization			0.11 (0.04, 0.27)	< 0.001
Inadequate notice about immunization outreach visit to community adjusted for Maternal Age + vaccinator conduct outreach immunisation + Parity (above 6 children) + Health facility too far and no transportation to going for immunization +child card availability			0.12 (0.04, 0.31)	< 0.001
Inadequate notice about immunization outreach visit to community adjusted for Maternal Age + vaccinator conduct outreach immunisation + Parity (above 6 children) + Health facility too far and no transportation to going for immunization +child card availability +Vaccinator not happy for coming regularly to the community			0.14 (0.05, 0.37)	< 0.001
**Churches discussed immunization importance:**				
Churches discussed immunization importance adjusted for county	7.42 (4.28,12.82)	< 0.001	2.42 (1.16, 5.01)	0.018
Churches discussed immunization importance adjusted for county + Health facility too far and no transportation to go for immunisation			1.95 (0.92, 4.13)	0.081
Churches discussed immunization importance adjusted for county + Health facility too far and no transportation to go for immunisation + Are you parent or guardian			2.02 (0.87, 4.65)	0.099
Churches discussed immunization importance adjusted for county + Health facility too far and no transportation to go for immunisation + Are you parent or guardian + Vaccinator visit during family livelihood business			1.82 (0.77, 4.24)	0.169
Churches discussed immunization importance adjusted for county + Health facility too far and no transportation to go for immunisation + Are you parent or guardian + Vaccinator visit during family livelihood business + Not completed all the immunization for my child, not informed to come back			1.77 (0.75, 4.18)	0.191
Churches discussed immunization importance adjusted for county + Health facility too far and no transportation to go for immunisation + Are you parent or guardian + Vaccinator visit during family livelihood business + Not completed all the immunization for my child, not informed to come back + Functional facility type in the area			1.93 (0.80, 4.63)	0.139
Churches discussed immunization importance adjusted for county + Health facility too far and no transportation to go for immunisation + Are you parent or guardian + Vaccinator visit during family livelihood business + Availability of children immunization card			2.19 (0.87, 5.47)	0.094
Churches discussed immunization importance adjusted for county + Health facility too far and no transportation to go for immunisation + Are you parent or guardian + Vaccinator visit during family livelihood business + Availability of children immunization card + Frequent provision of immunisation at health facility			2.26 (0.89, 5.72)	0.085
Churches discussed immunization importance adjusted for county + Health facility too far and no transportation to go for immunisation + Are you parent or guardian + Vaccinator visit during family livelihood business + Availability of children immunization card + Frequent provision of immunisation at health facility + Inadequate notice about immunization outreach visit to community			2.19 (0.82, 5.82)	0.115
Churches discussed immunization importance adjusted for county + Health facility too far and no transportation to go for immunisation + Are you parent or guardian + Vaccinator visit during family livelihood business + Availability of children immunization card + Frequent provision of immunisation at health facility + Inadequate notice about immunization outreach visit to community + Health Facility conduct routine immunisation outreach			2.09 (0.78, 5.59)	0.141
**Community discussed immunization importance:**				
Community discussed immunization importance: adjusted for Churches discussed immunization importance	7.76 (4.50,13.36)	< 0.001	3.62 (1.42, 9.18)	0.007
Community discussed immunization importance: adjusted for Churches discussed immunization importance + Availability of children immunization card			2.22 (0.85, 5.80)	0.103
Community discussed immunization importance: adjusted for Churches discussed immunization importance + Availability of children immunization card + Health facility too far and no transportation to going for immunization			2.22 (0.85, 5.74)	0.099
Community discussed immunization importance: adjusted for Churches discussed immunization importance + Availability of children immunization card + Health facility too far and no transportation to going for immunization + Are you Parent/guardian of the child			1.94 (0.74, 5.07)	0.178
Community discussed immunization importance: adjusted for Churches discussed immunization importance + Availability of children immunization card + Health facility too far and no transportation to going for immunization + Are you Parent/guardian of the child + Functional health facility types			1.38 (0.47, 3.97)	0.551
Community discussed immunization importance: adjusted for Churches discussed immunization importance + Availability of children immunization card + Health facility too far and no transportation to going for immunization + Are you Parent/guardian of the child + Functional health facility types + Inadequate notice about immunization outreach visit to community			1.13 (0.36, 3.43)	0.836
Community discussed immunization importance: adjusted for Churches discussed immunization importance + Availability of children immunization card + Health facility too far and no transportation to going for immunization + Are you Parent/guardian of the child + Functional health facility types + Inadequate notice about immunization outreach visit to community + Vaccinator visit during family livelihood business			1.13 (0.37, 3.364)	0.829
Community discussed immunization importance: adjusted for Churches discussed immunization importance + Availability of children immunization card + Health facility too far and no transportation to going for immunization + Are you Parent/guardian of the child + Functional health facility types + Inadequate notice about immunization outreach visit to community + Vaccinator visit during family livelihood business + Not completed all the immunization for my child, not informed to come back			1.23 (0.40, 3.71)	0.711
Community discussed immunization importance: adjusted for Churches discussed immunization importance + Availability of children immunization card + Health facility too far and no transportation to going for immunization + Are you Parent/guardian of the child + Functional health facility types + Inadequate notice about immunization outreach visit to community + Vaccinator visit during family livelihood business + Not completed all the immunization for my child, not informed to come back + Parity (above 6 children)			1.18 (0.38, 3.59)	0.777
Community discussed immunization importance: adjusted for Churches discussed immunization importance + Availability of children immunization card + Health facility too far and no transportation to going for immunization + Are you Parent/guardian of the child + Functional health facility types + Inadequate notice about immunization outreach visit to community + Vaccinator visit during family livelihood business + Not completed all the immunization for my child, not informed to come back + Parity (above 6 children) + Frequent provision of immunisation at health facility			1.24 (0.39, 3.84)	0.713
Community discussed immunization importance: adjusted for Churches discussed immunization importance + Availability of children immunization card + Health facility too far and no transportation to going for immunization + Are you Parent/guardian of the child + Functional health facility types + Inadequate notice about immunization outreach visit to community + Vaccinator visit during family livelihood business + Not completed all the immunization for my child, not informed to come back + Parity (above 6 children) + Frequent provision of immunisation at health facility + Health facility conduct routine immunisation outreaches			1.29 (0.40, 4.05)	0.665

Model 1: Association is adjusted for Maternal Age + parity + parent/guardian + child availability card

Model 2: Association is adjusted for immunisation benefit + community discussed + church discussed + Vaccinator visit during family livelihood business + BHWs + Health facility too far and no transportation to go for immunization

Model 3: Association is adjusted for Functional facility type in the area + Health facility conduct routine immunization outreach services + vaccinator not happy + did not inform to come back + frequent provision of immunisation at the health facilities + inadequate notice + health facility conduct routine immunisation

^#^means it is not statistically significant (*p* value > 0.05)

The findings were further supported by the results in the multilevel multivariable logistic regression models that adjusted for potential confounders in a 3-multilevel model - maternal and child characteristics (model 1a), community related variables (model 2b) and state health systems variables (model 3c) of Table 4. The study found that children cared by mothers or guardians with 6 and more children were 60% less likely to be off track with immunization than those with 1 child. The odds of children being off track with immunization increased for children whose mothers reported that immunization was essential for child growth compared to protection against diseases (AoR 7.45, 95% CI 2.06–26.87) (see Table 4). Children whose mothers reported that vaccinators did not visit during family business and livelihoods and had no means of getting to the health facility were 57 and 83% less likely to be off track within immunization (see Table 4). The chances of being off track with immunization increased among children whose mother reported that vaccinators did not conduct outreach services (AoR 7.21, 95%CI 3.82, 13.5), and lack of discussion by the community (AoR 8.01, 95% CI 4.02, 15.92) and churches (AoR 7.62, 95% CI 3.90, 14.87) on the importance of immunization (see Table 4).

## Discussion

This study analyzed the factors that influence the likelihood of children aged 0–23 living in South Sudan to be off track with their routine immunization schedule. We found that 75.5% of the participants were off track and that the percentage of off track status increased in subsequent vaccine doses. Findings similar to this were reported in a study in Ethiopia that showed decreasing immunization coverage in subsequent vaccine doses [28]. Our study found that the high proportion of off track children was driven mainly by community and state-level variables. A child who did not attend a church that discussed immunization importance was more than 2 times more likely to be off track with immunisation than children who went to a church that discussed immunization importance. A child whose community leaders did not discuss immunization importance was more than 3 times more likely to be off track than a child whose community leader did. Additionally, the probability of a child being off track was reduced by 85% when mothers and caregivers were given adequate notice about immunization outreach. This is linked to the finding that the conduct of outreach immunization was an influential factor as children without access to outreach immunization were 2 times more likely to be off track than children with access to outreach immunization. Furthermore, children who did not have vaccinators coming directly to the family livelihood business were about 70% less likely to be off track. The perceptions of mothers and caregivers was also seen to be an influential factor as children were more than 4 times more likely to be off track if they had mothers who believed that the benefit of immunization was more towards child growth than protection against disease. Additionally, children who had 6 siblings or more were up to 60% less likely to be off track compared to being an only child. In addition to community factors, we also noted that geographical factors influenced the likelihood of children being on track with their immunization in conflict-prone parts of South Sudan. This was supported by the finding that living close to health facilities and having transportation reduced a child’s chances of being off track by 85%.

**Fig. 3:**
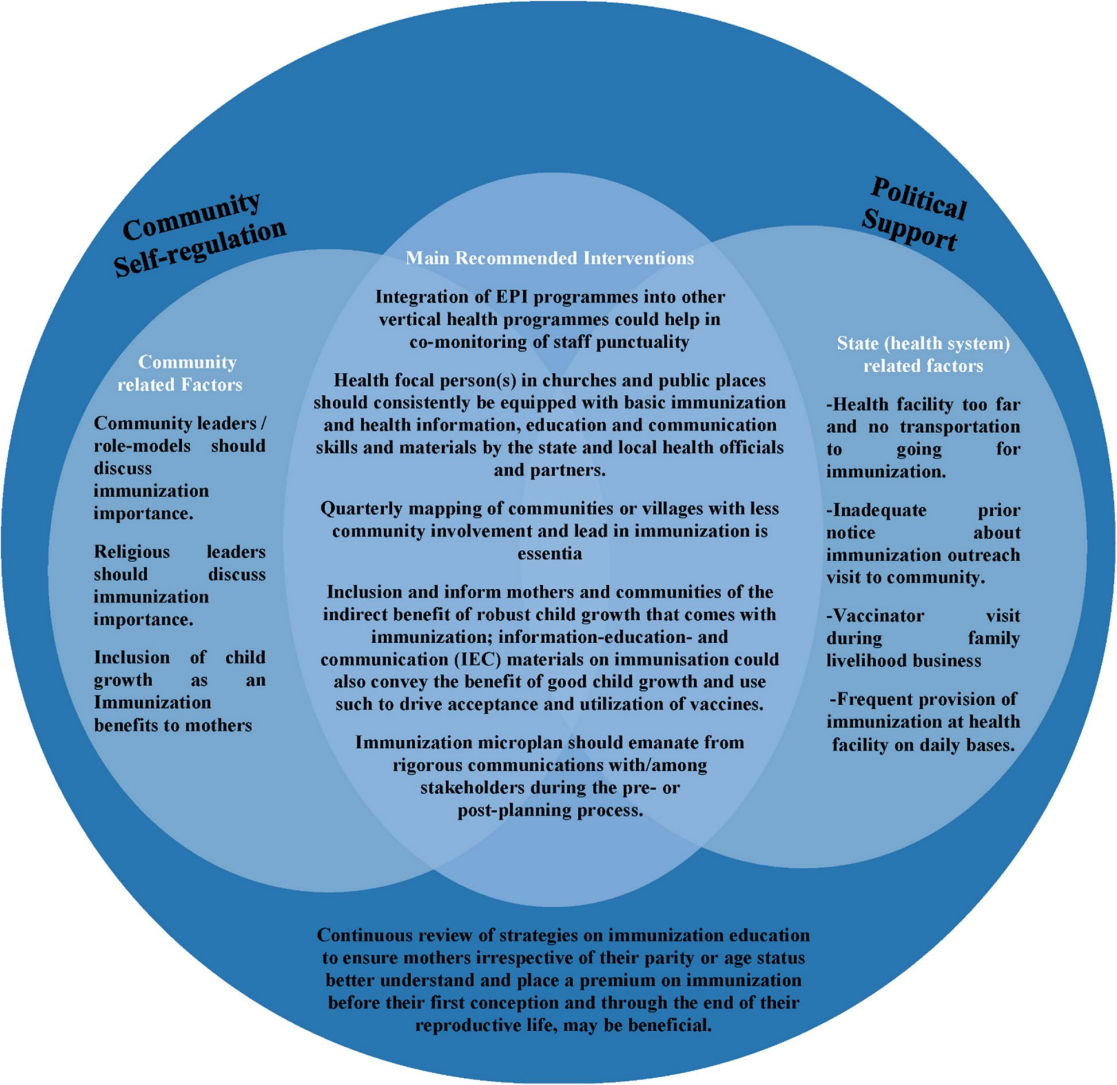
Key Points To Tackle The Predisposing Factors That Affects Children Living in South Sudan From Being on Track With Their Routine Immunization Schedule

Discussion about the benefits of Immunization in Churches was found to be a strong predictor of being off track with immunization. This finding highlights the role religious organizations play as influential platforms for educating the public and communicating the benefits of immunization to their worshippers. This finding contrasts with findings from a study in Nigeria which reported that religious leaders were reluctant to recommend vaccination to their followers on behalf of health authorities [29]. This reluctance to promote immunization in religious organizations could be related to the belief of some religious leaders that vaccination is against the ‘will of God’ as was reported by a study in Benin [30]. Although our study did not investigate whether and to what extent churches and worship centers are involved in immunization activities, we recommend that there is the need to review existing policies to ensure that religious organizations are involved in the planning and implementation of immunization programmes, and the monitoring of vaccine utilization at the community level. Similarly, a child whose community leader did not discuss immunization importance was over 3 times more likely to be off track than a child whose community leader did. This finding justifies the need for the active involvement of community leaders in driving immunization programme success. This finding justifies the need for the active involvement of community leaders in driving immunization programme success. The finding corroborated a study in Nigeria [31] which reported that efforts of community leaders and other community-level factors positively influenced the knowledge of mothers on immunization benefits. This finding also corroborated a study which focused on community engagement, routine immunization, and polio legacy in Northern Nigeria, a similar conflict context area with a long history of poor immunization and health performance [32]. Another study showed that the quality and volume of immunization and health information available in mothers’ social environment influenced the uptake of immunization services [33]. Based on the findings of our study, it can be deduced that the quality and volume of immunization information can be promoted through community leaders. This makes it imperative for community leaders to be more involved in promoting immunization education considering that in the absence of formal education facilities, community leaders can advance community health education, especially for girls and women. We observed that the probability of a child being off track was reduced by 85% when mothers and caregivers were given adequate notice prior to immunization outreach. This finding highlights the benefit of optimizing immunization outreaches and the provision of adequate notice to mothers and caregivers, and encourages enhanced application of immunization outreaches within our study population. The optimization of immunization outreaches was supported by a WHO report that identified inadequate prior notice to immunization as one of the barriers to vaccine utilization [34]. In order to achieve an effective immunization outreach program, a micro-plan that involves rigorous communications with community stakeholders during the pre- or post-planning process should be implemented. Existing EPI micro-plans should be reviewed for areas where services are not optimized to reflect what works best for all stakeholders. The micro-plan developed in consultation with the benefiting communities, should have social mobilization well integrated in it as outreach activity is partially determined by the robustness of social mobilizers’ commitment (see Fig. 3). Furthermore, the commitment and quality of social mobilizers should be reestablished to ensure communities, government and the donors are not being short-changed with inadequacy or non provision of services. Since social mobilizers are engaged as a matter of policy in implementing immunization outreaches, immunization programme managers should ensure that mobilizers have good knowledge of the area they are to cover, comfortable to work in these insecure areas and are accepted by their assigned community. Otherwise, the use of multiple mobilizers may be adopted by communities with wide land mass or with security challenges. Social mobilizers and EPI Vaccinators providing outreach services should be regularly supported, mentored and monitored (SMM) to improve integration and deliver quality immunization services. In this regard, we recommend not more than two to three immunization sessions per week with more days available for adequate social mobilization. Furthermore, as seen in the study’s results, children who did not have vaccinators during family livelihood business were about 70% less likely to be off track. In a study conducted in Burkina Faso, mothers who gave more concern to their business were found not to have their children with full vaccination [35]. The finding indicates that mothers/caregivers are likely to prioritize their businesses over the immunization of their children since the timing of immunization clashes with their business hours. This reiterates the usefulness of a well planned and coordinated vaccination plan with input from various interests including the working mother population.

This study observed that health facilities frequently conducting routine (static) immunization services on a daily basis were influential in reducing the amount of children off track with immunization. Policy on immunization in South Sudan advises the implementation of routine immunization outreach across communities outside the 5 km radius of a health facility [1, 36, 37]. Considering the need for improvement of immunization success in countries like South Sudan, continuous vaccine availability and punctuality of EPI vaccinators at health facilities, creation and sustainability of more outreach posts with regular supplementary immunisation activities in hard-to-reach areas could translate into higher vaccine uptake. The above mixed intervention should be evaluated regularly to ensure communities, government and the donors get appropriate service and value for immunisation programmes. While promoting staff retention is important to achieving sustained progress, vaccinators who are found deficient with low-commitment should be given refreshers’ training while those with repeated underperformance should be let go in order to engage committed ones. We also recommend the continual usage of performance incentives to pay vaccinators for services rendered; and for every falsehood identified in the immunization accountability conducts and reports, sanctions should be designed. To promote the effectiveness of performance based incentives, an improved system of accountability has to be put in place. There is an urgent need for South Sudan to put forward a clear policy and implementable strategies on training of vaccinators, and/or who should be responsible for vaccination delivery at health facilities and within communities. State immunization performance monitoring and evaluation strategy must be reviewed at least twice in a year, and interventions should be fashioned to model collaborative, iterative and inclusive and targeted problem solving approaches. According to a unicef report, a ‘one-size-fits-all’ approach in this changing world is no longer appropriate [38]. Nevertheless, analysis of data available from DHIS2 have shown more vaccination still takes place during outreaches activities for most counties in south Sudan [1, 39].

When it came to mothers/ caregivers and their thoughts towards immunization, children had a higher odds of being off track if they had mothers who believed the benefits of immunization is for child growth compared to protection against diseases. Extensive evidence has also consistently listed child protection from diseases as a primary benefit from complete vaccination of children [40, 41]. Interestingly, our sampled mothers place higher value on good child growth as the benefit of immunization rather than protection against diseases to their children. In this regard, we recommend educating caregivers on the benefits of immunization as it has a great influence on immunization uptake (see Fig. 3).

In this study, it was noted that mothers who had 6 or more children had a lower odds of being off track compared to those whose mothers had one child. Studies conducted in Liberia and another in Kenya showed a reduced chance of completion of vaccination for children with 2–3 siblings but the chance of completion increased for children with 4 siblings and above, when compared to one child only [42]. Studies conducted in Liberia and another in Kenya showed a reduced chance of completion of vaccination for children with 2–3 siblings but the chance of completion increased for children with 4 siblings and above, when compared to one child only. This finding is contrary to the reports from Eastern Ethiopia (OR 3.55, CI: 1.32–9.58) [43] and West Africa that showed higher odds of defaulting from immunization with mothers of higher parity [44].

Not only was the outcome dependent on community factors, but also geographical factors in that living close to health facilities and having transportation reduced a child’s chances of being off track by 85%. This finding demonstrates the importance of health equity and increasing immunization access to hard-to-reach and far communities. This study finding is in line with a study conducted in Kenya that showed mothers residing about > 5 km from a health facility (OR 1.6, CI1.1–3.1) were more likely to default from completing vaccination [42]. Contrarily, studies conducted in Mali (OR:0.6, CI: 0.4–0.8) and Niger (OR: 0.8, 0.6–1.0), showed that living closer to health facilities was not associated with complete vaccination of children [44]. Activities that positively influence health seeking behaviour and immunization information sharing should be promoted to optimise immunization service utilization. While it may be a good recommendation based on the study finding for government and partners to establish more health facilities considering its potential benefits to immunization and population health generally, location and localization of health facilities are governed by other factors including population and resources which may not immediately support that. Hence, a proper assessment of the health (facility) needs is highly recommended. Political influence in the positioning of health facilities should be minimized if it cannot be completely stopped. Robust implementation and coordination between the health facility, outreach and mobile immunisation services should be urgently considered as they could be ideal in reducing the amount the caregivers use in transportation costs.

In all, this study has revealed that improving immunization coverage requires strengthening community health systems, and this should remain a key priority for government and major health partners.

### Study limitation

This cross-sectional study is reported in accordance with the 22- item checklist that are considered highly essential by the STROBE- strengthening the Reporting of Observational Studies in Epidemiology statement; however, this study would have had more improved quality if a well-structured survey with a variety of variables. In addition, we acknowledge bias in the study questionnaire like social desirability bias as a result of mothers who may have given inaccurate answers in an effort to be viewed more favorably as well as interviewers’ bias or influence would have occurred from the integrated community case management {(ICCM) or Boma health} workers with the tendency of the interviewer to obtain answers that support preconceived notions, while writing on behalf of mothers who were unable to read and write. Infact, bias as a result of a non- standardized measurement of conflict or risk intensity among these counties which could have had an association with the immunisation uptake coverage in these respective counties at the time of the study. This study could not assess the timeliness of vaccine uptake by children, and also recommend further studies with a larger sample size with more equal sampling across the counties for more comparable conclusions. We also admit differential misclassification of caregivers or mothers may have occurred with adopted children in the class of mothers with a higher parity; in addition to that, excluding children of mothers who reported the child is being immunised but with untraceable immunisation data records from the study could also have resulted in selection bias and non-differential misclassification and could have underestimated the proportion of the groups of children being on and off track with their immunisation schedule. Lastly, we also acknowledge that residual confounding may have occurred in the grouping of mother and child’s ages, parity, distance to health facility.

## Conclusion

This study showed that a high proportion of children are off track with their immunization schedule and this is mainly driven by factors linked to immunization service delivery mechanisms at community and state levels. These factors include but are not limited to sub-optimal communication about immunization by religious organizations and community leaders, inadequate notice of outreach immunization to mothers/care-givers, limited reach of of outreach vaccination, the perceptions of mothers/care-givers’ towards immunization, and travel distance to health facilities. Based on the findings of our study, we recommended potential interventions to improve child immunization uptake in South Sudan. The findings of this study are not likely to be transferable to a non-conflict context, nevertheless, we strongly recommend regular evaluation of the performance of health system vaccine delivery at the community level.

## Supplementary Information

**Additional file 1: Table 1.** Current immunization schedule route of administration.

**Additional file 2.**

## Data Availability

The datasets used and/or analysed during the current study are available from the corresponding author on reasonable request.
