# Supplementary material for: Are children on track with their routine immunization schedule in a fragile and protracted conflict state of South Sudan? A community-based cross-sectional study
Source: BMC Pediatr. 2022 Mar 21;22:147. doi: 10.1186/s12887-022-03213-5 (PMC8935713; doi:10.1186/s12887-022-03213-5)
Supplement: Supplementary file 1 — Additional file 1: Table 1. Current immunization schedule route of administration. [file 12887_2022_3213_MOESM1_ESM.docx]

**Additional file**

Table 1: Current immunization schedule route of administration

| **Antigen** | **Minimum age** | **Dose to be administered** | **Route of administration** |
| --- | --- | --- | --- |
| OPV 0 | At birth | 2 drops | By mouth-use provided dropper |
| BCG | At birth | 0.05 ml (infants 0-11 months) | Intradermal (within the skin): left arm |
|  |  | 0.01 ml (children above 11 months) |  |
| PENTA 1 | 6 weeks | 0.05 ml | Intramuscularly in the upper outer part of the thigh |
| OPV 1 | 6weeks | 2 drops | By mouth-use provided dropper |
| PENTA 2 | 10 weeks | 0.05 ml | Intramuscularly in the upper outer part of the thigh |
| OPV 2 | 10 weeks | 2 drops | By mouth-use provided dropper |
| PENTA 3 | 14 weeks | 0.05 ml | Intramuscularly in the upper outer part of the thigh |
| OPV 3 | 14 weeks | 2 drops | By mouth-use provided dropper |
| IPV | 14 weeks | 0.05 ml | Intramuscularly in the upper outer part of the thigh |
| Measles | 9 months | 0.05 ml | Measles vaccine is given subcutaneously in the outer aspect of the deltoid muscle (Lt) at 9 months. However, during supplementation activities, the target group may increase to 15 years as advised by WHO |
