# Supplementary material for: Are children on track with their routine immunization schedule in a fragile and protracted conflict state of South Sudan? A community-based cross-sectional study
Source: BMC Pediatr. 2022 Mar 21;22:147. doi: 10.1186/s12887-022-03213-5 (PMC8935713; doi:10.1186/s12887-022-03213-5)
Supplement: Supplementary file 2 — Additional file 2. [file 12887_2022_3213_MOESM2_ESM.docx]

**PREDISPOSING FACTORS FOR IMMUNIZATION DEFAULTING AMONG UNDER-23 MONTHS IN HARD TO REACH COMMUNITIES IN LAKES SOUTH SUDAN**

*(This questionnaire is to be administered to the mother/guardian of the child observed to have defaulted immunization)*

**Section A:** Name of the defaulted child…………………………..………………………………………….…Age……………(months)

Name of mother/guardian ……………………………..………………………..……….….. Age of mother/guardian…..……… (yrs)

Educational level………………….………….. Marital Status…………………………………….. Religion…………….………………………

No of children:………………. Village/Boma……………….……………..……………………...………… County…………………….………

Child Health Card: Available/Not Available…………………….………… Child’s gender: Male/female………………………

Immunization type taken to date: 1 ……………….…... … 2………………..……….3…………………..……...4……………………….….

5………………………………6………………………………………7………….…..…………………8……………………………9………………………….

Missed Immunization: 1………………………………..……… 2…………………….…………………… 3…………………………………….…...

**Section B:** Awareness of Immunization and Immunization services delivery

Is there any functional health facility in the area? Yes or No…………… / Type (PHCU/PHCC/HOSPITAL)……………..………………………...

How far is the nearest health facility to the child’s home?…………………….……(in Km)

Does the health facility provide immunization (EPI) services? Yes or No……………… Daily? Yes/No…………………

Does the health facility conduct routine immunization outreach services? Yes or No…………………..……..……….……

Does the health facility conduct mobile immunization outreach services? Yes or No………….……………………………

Do you think immunization is good for your child’s health? Yes or No………… Benefits:.……………..………………..……

**Section C:** Possible reason for the child defaulting immunization? **Kindly Tick appropriate response**

1. The EPI Vaccinator/health care workers do not reach their community during immunization outreach **YES / NO**
2. The time the Vaccinators usually visit is the time I go for my business to earn for family livelihood? **YES / NO**
3. The home is considered too far from the health facility/no transportation to go for immunization **YES / NO**
4. I do not like the attitude/behavior of the Vaccinators /healthcare workers/ Vaccinators are abusive **YES / NO**
5. Vaccinators/health care workers usually ask for money any time they visit the health facility for service **YES / NO**
6. Vaccinators are not happy coming to their community/not motivated to come regularly **YES / NO**
7. I do not know I have not completed all the immunization for my child/I was not informed to come back **YES / NO**
8. Inadequate notice/pre-information about the immunization outreach visit to their community **YES / NO**
9. The cry/pain my child had during the past immunization was too much /fear of the side effect **YES / NO**
10. I do not want my girl child to receive immunization **YES / NO**
11. I do not want my male child to receive immunization.  **YES / NO**

If j and/or k is yes, why?...............................................................................................................................

1. Does the child have relocation history in the past 23 months? **YES / NO**
2. How many times have the BHW visited your household? ________________________
3. Parent / Guardian of the child? **YES / NO**
4. Any other reason:……………………………………………………………………………………………………………………………….…

**Informed Consent Form**

**Introduction**

We are conducting a research titled: **PREDISPOSING FACTORS FOR IMMUNIZATION DEFAULTING AMONG UNDER-23 MONTHS IN HARD TO REACH COMMUNITIES IN LAKES SOUTH SUDAN**

To carry out this study, I will be asking you and other respondents like you a number of questions which will take about 5-20 minutes to complete. Please note that all information collected through this questionnaire will be kept confidential.

The outcome of this research will be useful in identifying barriers to immunization in your community, which will be used as a form of publication and advocacy for the government of South Sudan and other developing countries for adoption in their immunization strengthening and reform.

There is no risk in taking part in this survey and you have the right to **decline** participation at any time. Your name will **NOT** be captured on the forms I use to write down your response to questions or the report/publication of this study. Your participation in this research is entirely voluntary. Please make sure any doubts you have are cleared before you agree to take part.

**Consent to Participate**

I have read the description of the research and also talked it over with the Expert / Researcher to my satisfaction. I understand that my participation is voluntary.

I know enough about the purpose, methods, and benefit attached to the research study to judge that I want to take part in it. I understand that I may freely stop being part of this study at any time.

Name or Initials of Respondent. ……………………………………………………………….

Signature....................................... Date.................................

Name of Interviewer (Only BHI workers /Supervisors) ………………………………………………………………..

Signature ...................................... Date**..................................** Questionnaire ID..................

This research has been approved by the Ethics Committee of Lakes State Ministry of Health.

**Thank you**
